# Pre-Clinical Development of a Potent Neutralizing Antibody MW3321 With Extensive SARS-CoV-2 Variants Coverage

**DOI:** 10.3389/fphar.2022.926750

**Published:** 2022-07-08

**Authors:** Wen Jiang, Zherui Zhang, Yuhe Zhu, Ben Chen, Chunying Gu, Zhiyan Liu, Xukai Zhang, Hualong Xiong, Yanan Zhang, Bin Zheng, Rongjuan Wang, Shasha Jiao, An Wang, Tianying Zhang, Jinchao Zhang, Shuang Wang, Bo Zhang, Gang Li, Xun Gui

**Affiliations:** ^1^ Mabwell (Shanghai) Bioscience Co., Ltd., Shanghai, China; ^2^ Key Laboratory of Special Pathogens and Biosafety, Wuhan Institute of Virology, Center for Biosafety Mega-Science, Chinese Academy of Sciences, Wuhan, China; ^3^ State Key Laboratory of Molecular Vaccinology and Molecular Diagnostics, National Institute of Diagnostics and Vaccine Development in Infectious Diseases, School of Public Health, Xiamen University, Xiamen, China; ^4^ Beijing Kohnoor Science and Technology Co., Ltd., Beijing, China

**Keywords:** SARS-CoV-2, neutralizing antibody, escape mutation, antibody therapeutics, safety profile

## Abstract

Since the outbreak of the coronavirus disease 2019 (COVID-19) pandemic, several variants of the severe acute respiratory syndrome coronavirus 2 (SARS-CoV-2) have emerged and have consistently replaced the previous dominant variant. Therapeutics against variants of SARS-CoV-2 are urgently needed. Ideal SARS-CoV-2 therapeutic antibodies would have high potency in viral neutralization against several emerging variants. Neutralization antibodies targeting SARS-CoV-2 could provide immediate protection after SARS-CoV-2 infection, especially for the most vulnerable populations. In this work, we comprehensively characterize the breadth and efficacy of SARS-CoV-2 RBD-targeting fully human monoclonal antibody (mAb) MW3321. MW3321 retains full neutralization activity to all tested 12 variants that have arisen in the human population, which are assigned as VOC (Variants of Concern) and VOI (Variants of Interest) due to their impacts on public health. Escape mutation experiments using replicating SARS-CoV-2 pseudovirus show that escape mutants were not generated until passage 6 for MW3321, which is much more resistant to escape mutation compared with another clinical staged SARS-CoV-2 neutralizing mAb MW3311. MW3321 could effectively reduce viral burden in hACE2-transgenic mice challenged with either wild-type or Delta SARS-CoV-2 strains through viral neutralization and Fc-mediated effector functions. Moreover, MW3321 exhibits a typical hIgG1 pharmacokinetic and safety profile in cynomolgus monkeys. These data support the development of MW3321 as a monotherapy or cocktail against SARS-CoV-2-related diseases.

## Introduction

After its emergence in December 2019, the ongoing COVID-19 pandemic caused by severe acute respiratory syndrome coronavirus 2 (SARS-CoV-2), has caused devastating consequences to human health and the global economy. As of 22 April 2022, SARS-CoV-2 has spread to more than 200 countries/territories and resulted in more than 6.2 million deaths (World Health Organization). Effective countermeasures are urgently needed to control the pandemic and protect vulnerable populations.

Coronaviruses are zoonotic pathogens responsible for several epidemics and a pandemic in the past two decades (Du et al., 2009; Li et al., 2015; Wang et al., 2016). All three highly pathogenic coronaviruses belong to the betacoronavirus genus: SARS-CoV-2 and SARS-CoV cluster within the sarbecovirus subgenus and originated in bats, whereas Middle East respiratory syndrome coronavirus (MERS-CoV) belongs to the merbecovirus subgenus and is transmitted to humans through dromedary camels. Cellular entry of SARS-CoV-2 and SARS-CoV is mediated by the viral spike glycoprotein, which forms trimeric spikes on the viral surface. Like SARS-CoV, the receptor-binding domain (RBD) of SARS-CoV-2 spike protein is responsible for engaging the angiotensin-converting enzyme 2 (ACE2) receptor on the host cell surface and mediating cell-virus membrane fusion by the class I fusion mechanism (Hoffmann et al., 2020; Ou et al., 2020). The immune response to the SARS-CoV-2 virus involves a combination of cell-mediated immunity and antibody production. It remains unknown if natural immunity to the SARS-CoV-2 virus will be long-lasting in recovered individuals. One of the concerns relates to the virus’ continual accumulation of mutations, which may alter the spectrum of viral antigenicity and cause reinfection by mutant strains of the virus. These variant strains may harbor mutations that ultimately enhance viral recognition and infection into host cells, thereby increasing infectivity and/or pathogenicity. The WHO has named Alpha (B.1.1.7, December 2020) (Tang et al., 2021), Beta (B.1.351, January 2021) (Tegally et al., 2021), Gamma (P.1, January 2021) (Sabino et al., 2021), Kappa (B.1.617.1, 2020) (Ferreira et al., 2021), Delta (B.1.617.2, May 2021) (Mlcochova et al., 2021), Lambda (C.37, 2021) (Romero et al., 2021), and other variants, several of which show potential for increased transmissibility, increased virulence, or vaccine resistance.

One promising approach to combat the COVID-19 pandemic involves the development of antiviral antibodies as treatment or prevention therapeutics (Dougan et al., 2021; Gupta et al., 2021; Weinreich et al., 2021). Anti-SARS-CoV-2 neutralizing mAbs that target the spike protein have been shown to have clinical benefits in treating SARS-CoV-2 infection both as post-exposure prophylaxis or pre-exposure prophylaxis. Four anti-SARS-CoV-2 mAb products have received Emergency Use Authorizations (EUAs) from the Food and Drug Administration (FDA). Bamlanivimab plus etesevimab, casirivimab plus imdevimab (REGEN-COV), and sotrovimab received EUAs for the treatment of mild to moderate COVID-19 in nonhospitalized patients with laboratory-confirmed SARS-CoV-2 infection who are at a high risk for progressing to severe disease and/or hospitalization; and the FDA has issued an EUA for tixagevimab plus cilgavimab (Evusheld), a long-acting anti-SARS-CoV-2 mAb combination. The EUA allows this combination to be used as SARS-CoV-2 pre-exposure prophylaxis for individuals who do not have SARS-CoV-2 infection. However, the emerging SARS-CoV-2 variants escape the neutralization of some potent neutralizing antibodies, diminish the effectiveness of the approved vaccines, and reduce the efficacy of the existing antibody cocktail treatment (Wang et al., 2021a; Wang et al., 2021b; Hoffmann et al., 2021; Planas et al., 2021).

We previously reported the isolation of a SARS-CoV-2 Spike RBD-targeting mAb, MW06 (Drug Code: MW3321), which has cross-reactivity with SARS-CoV (Jiang et al., 2021; Wang et al., 2022). Here in this work, we identify the neutralization potency of MW3321 to a series of SARS-CoV-2 variant strains in pseudoviral neutralization assays. *In vivo* studies show that MW3321 sharply reduces viral load in hACE2-transgenic mice challenged with either wild-type or Delta SARS-CoV-2 strains. Importantly, MW3321 is much more resistant to escape mutation compared with another clinical staged SARS-CoV-2 neutralizing mAb, MW3311, in the pseudoviral escape mutants screening system. Moreover, a typical hIgG1 pharmacokinetic and safety profile was observed in cynomolgus monkeys. Overall, these data support the development of MW3321 for further clinical use.

## Materials and Methods

### Cells and Viruses

Huh-7 (Institute of Basic Medical Sciences CAMS, 3111C0001CCC000679) cells and Vero-E6 (ATCC, CRL-1586), and BHK21-hACE2 (Xiong et al., 2020) cells were maintained in high glucose DMEM (SIGMA-ALDRICH) supplemented with 10% FBS (GIBCO, 10099-141), penicillin (100 IU/ml), and streptomycin (100 μg/ml) in a 5% CO_2_ environment at 37°C. Replicating VSV pseudovirus carrying truncated spike protein of SARS-CoV-2, named VSV-SARS-CoV-2-Sdel18 virus, was packaged as previously described (Xiong et al., 2020). Nonreplicating SARS-CoV pseudovirus was prepared and provided by the Institute for Biological Product Control, National Institutes for Food and Drug Control.

### Escape Mutation Study

VERO-E6 cells were cultured in 3–6 × 10^6^ cells/6 cm cell culture plates and infected with a virus with the multiplicity of infection (MOI) of 0.01 when the cell density is up to 70%–90%. The indicated antibody was diluted with a 5% complete medium to the concentration of 20 μg/ml and an 80 μl/plate was added to the corresponding virus plate. After overnight incubation, the virus or antibody was supplied if the inhibition (%) was above 90% or below 30%, respectively. The supernatants were collected when the virus infection rate of cells was above 80% or the cytopathic rate was above 80%. For the following selections, the supernatants were repeatedly inoculated into VERO-E6 cells. Escaping virus amplification and titer determination were carried out with BHK21-hACE2 cells.

### Neutralization Assay

For the SARS-CoV-2 pseudoviral neutralization assay against different variants, 100 µl of mAbs at different concentrations were mixed with 50 µl supernatant containing ∼1000 TCID_50_ SARS-CoV-2 pseudovirus. The mixture was incubated at 37°C for 1 h, and supplied with 5% CO_2_. 100 µl of Huh-7/ACE2 (3 × 10^5^ cells/ml) was then added to the mixture of pseudovirus with mAbs for an additional 24-h incubation at 37°C. Luciferase detecting reagents (PerkinElmer and 6066769) were added, and the luciferase activity was measured using a microplate luminometer (Molecular Devices, SpectraMax L). A four-parameter logistic fit was performed with log-transformed sample concentration (μg/ml) as the abscissa, and neutralizing (%) as the ordinate. The concentration of 50% inhibition (IC50) was calculated using GraphPad Prism 8.2.1. For VSV-SARS-CoV-2 pseudoviral neutralization, BHK21-hACE2 cells were plated into a 96-well plate with 5 × 10^4^ cells overnight. The virus was diluted as 0.05 MOI, and incubated with serially diluted indicated antibody for 1 h at 37°C. The supernatant of BHK21-hACE2 cells was drawn and discarded and the virus-antibody mixture was added with 80 μl/well. After incubation at 37°C for 12 h, the number of positive cells was recorded by laser confocal high-content microscopy photography. The neutralization inhibition rate of antibody against escape virus strains = (number of GFP-positive cells in MOCK wells—number of GFP-positive cells in experimental wells)/number of GFP-positive cells in MOCK wells × 100%, The IC_50_ was calculated using GraphPad Prism 8.2.1.

### Antibody-Dependent Cellular Cytotoxicity Assay

SARS-CoV-2 spike protein-expressing CHO-K1 cells (CHO-K1/SARS-CoV-2-S) were seeded into a 96-well plate with 5000 cells/well followed by overnight incubation. MW3321 or IgG1 isotype control were serially diluted. The supernatant was removed and diluted antibodies were added (50 μl/well) to the cells and incubated for 1 h. PBMC was diluted to 5 × 10^6^ cells/ml and added to a 96-well plate at 50 μl/well. After an additional overnight incubation at 37°C, the released LDH was detected with Cytotoxicity LDH Assay Kit-WST (DOJINDO, CK12) and measured using a microplate reader (Molecular Devices, SpectraMax i3x). %Cytotoxicity = (Experimental Signal–target cells signal–effector cells signal)/(Max signal–target cells signal) x 100%. A four-parameter logistic fit was performed with log-transformed sample concentration (ng/ml) as the abscissa, and %Cytotoxicity as the ordinate using GraphPad Prism 8.2.1.

### Complement-Dependent Cytotoxicity Assay

SARS-CoV-2 spike protein-expressing CHO-K1 cells (CHO-K1/SARS-CoV-2-S) were seeded into a 96-well plate with 5 × 10^4^ cells/well followed by overnight incubation. Antibodies were serially diluted. MabThera (Roche, H0205) on target Raji cells was used as the positive control. The supernatant was removed and the diluted antibody and 8% normal human serum complement protein (Quidel, A113) were added to the cells. After an additional overnight incubation at 37°C, the plate was then incubated with the dye Resazurin (Rhino Bio, QDY-002-D) for cell viability detection and measured using a microplate reader (Molecular Devices, SpectraMax i3x)*.* The percentage of cytotoxicity was calculated by following formula: %Cytotoxicity = 100% × (RLU_completment+cell_ – RLU_sample_)/(RLU_cell + complement_ – RLU_complement_). A four-parameter logistic fit was performed with log-transformed sample concentration (ng/ml) as the abscissa, and %Cytotoxicity as the ordinate using GraphPad Prism 8.2.1.

### Antibody-Dependent Cellular Phagocytosis Assay

SARS-CoV-2 spike protein-expressing CHO-K1 cells (CHO-K1/SARS-CoV-2-S) were seeded into a 96-well plate with 3000 cells/well followed by overnight incubation. MW3321 and REGN10987 (Hansen et al., 2020) were serially diluted. The supernatant was removed and added with 50 μl/well-diluted antibodies and incubated for 1 h. ADCP luciferase reporter cells (Jurkat/NFAT/CD32a-FcεRIγ) as effector cells, were diluted to 2.4 × 10^6^ cells/ml and added to a 96-well plate at 50 μl/well. After an additional overnight incubation at 37°C, luciferase detecting reagents (PerkinElmer, 6066769) were added and the luciferase activity was measured using a microplate luminometer (Molecular Devices, SpectraMax L). A four-parameter logistic fit was performed with log-transformed sample concentration (ng/ml) as the abscissa, and RLU as the ordinate using GraphPad Prism 8.2.1.

### *In Vivo* Efficacy of MW3321 in SARS-CoV-2 Infection Mouse Model

Animal studies were performed according to the procedures approved by the Chinese Academy of Sciences and complied with all relevant ethical regulations regarding animal research. Female H11-K18-hACE2 mice (6–8 weeks old) were inoculated with 10^5^ PFU of wild-type and Delta SARS-CoV-2 via the intranasal route. Indicated antibodies were administered 2 h after SARS-CoV-2 inoculation. Weights were monitored daily; mice were euthanized at 3 days post-infection (dpi) and lung tissue was collected. Uninfected mice were set as control. A plaque assay was used to measure the lung virus burden. The lung tissues were homogenized in a 1 ml DMEM medium. Homogenates were 10-fold serially diluted and applied to Vero-E6 cell monolayers in 24-well plates. Plates were incubated at 37°C for 1 h. Cells were then overlaid with 1% (w/v) methylcellulose. Plates were collected 4 days later by removing overlays and fixed with 1 ml staining buffer (3.7% methanol +1% crystal violet) per well overnight. Excess staining buffer was washed away with fluid water, and plaques were counted after drying.

### Pharmacokinetic and Safety Evaluation

For the pharmacokinetic study, eighteen (9 males and 9 females) naïve cynomolgus monkeys divided into 3 groups (6 animals/group, 3 males and 3 females) were enrolled in the study and administered with MW3321 at 5, 20, and 50 mg/kg by a single intravenous infusion. Blood sampling was performed at pre-dose, and 0.083, 4, 8, 24, 48,72, 96, 120, 168, 240, 336, 504, 672, 840, 1008, and 1176 h post-dosing. Serum concentrations of MW3321 were determined using a validated ELISA method by an independent laboratory (Jiangsu Tripod Preclinical Research Laboratories Co., LTD.). The main pharmacokinetic parameters were calculated using WinNonlin 8.1 software.

The safety profile of MW3321 was explored in a GLP-compliant 3-week repeat-dose toxicology study with a 3-week recovery period at doses of 40, 200, and 500 mg/kg in 40 cynomolgus monkeys (5/sex/group). The evaluations and related parameters included clinical observations, body weight, body temperature, food consumption, ophthalmology, ECG, clinical pathology, lymphocyte phenotyping, cytokines, circulating immune complex, organ weight and coefficient, histopathology, and safety pharmacology, local tolerance, immunogenicity, and toxicokinetic studies (Jiangsu Tripod Preclinical Research Laboratories Co., LTD.).

### Statistical Analysis

Data were analyzed with Prism 8.2.1 software (GraphPad Software, La Jolla, CA, United States) and were presented as mean ± SD. Unpaired t-tests were used to determine statistical significance when comparing two groups. A value of *p* < 0.05 was considered statistically significant.

## Results

### Broad Neutralization Potency of MW3321 Against SARS-CoV-2 Variants

**FIGURE 1:**
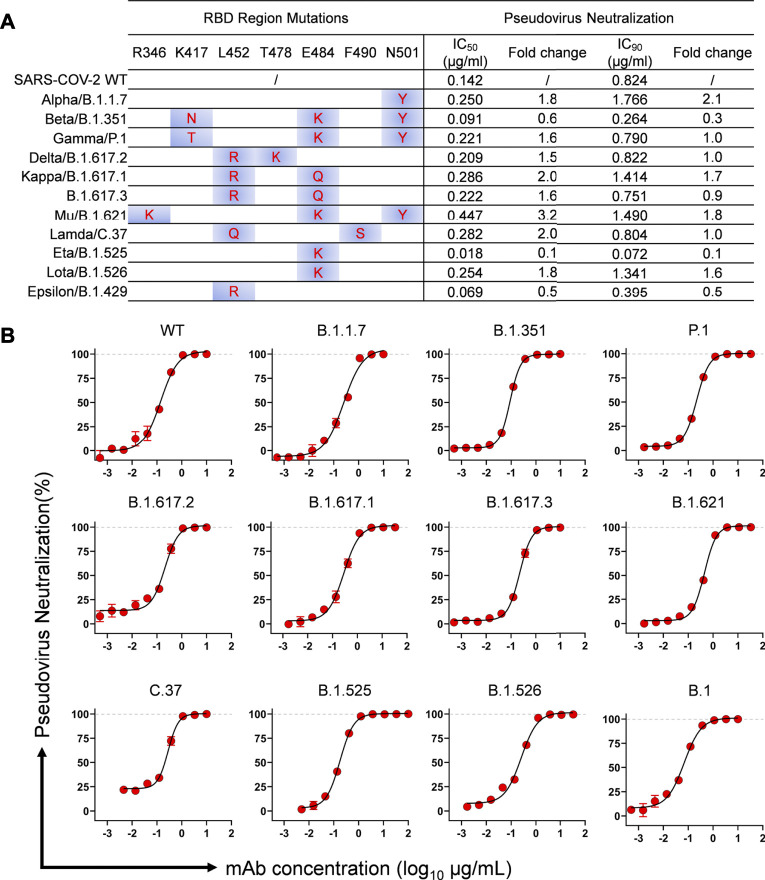
Neutralization activity of MW3321 against SARS-CoV-2 pseudovirus of different variants on Huh7/ACE2 cells. **(A)** Summary of the mutations within the RBD region and neutralization potency of MW3321. IC_50_ and IC_90_ are indicated. Fold change was calculated based on the IC_50_ or IC_90_ values of MW3321 to SARS-CoV-2 wild-type virus and variants. **(B)** Neutralization curves of MW3321 to SARS-CoV-2 variants. The data points are represented as mean ± SD with *n* = 3.

In our previous study, MW06 was identified to recognize a conserved epitope on spike RBD of SARS-CoV-2 and SARS-CoV (Jiang et al., 2021). To further evaluate the neutralization potency and breadth of MW3321, we assessed the neutralization potency of MW3321 to several VOCs and VOIs assigned by the WHO, which posed an increased risk to global public health via increased transmissibility or virulence, or decreased effectiveness of vaccines, diagnostic tools, and therapeutic medicines, in SARS-CoV-2 pseudoviral neutralization assays. The mutations in spike RBD of each variant was summarized in the left panel of Figure 1A. The mutation sites in these epidemic variants showed no overlap with the epitope of MW3321 (Jiang et al., 2021), which in retrospect revealed the conservation of the recognition epitope of MW3321. MW3321 exhibited potent neutralization activity against all these variants with IC_50_ values ranging from 0.02 μg/ml to 0.4 μg/ml, and IC_90_ from 0.07 μg/ml to 1.8 μg/ml (Figures 1A,B). Compared with the SARS-CoV-2 wild-type strain, MW3321 showed quite similar neutralization potency to other variants. MW3321 is therefore a broadly neutralizing mAb against SARS-CoV-2 variants.

### Prevention of SARS-CoV-2 Rapid Mutational Escape by MW3321

**FIGURE 2:**
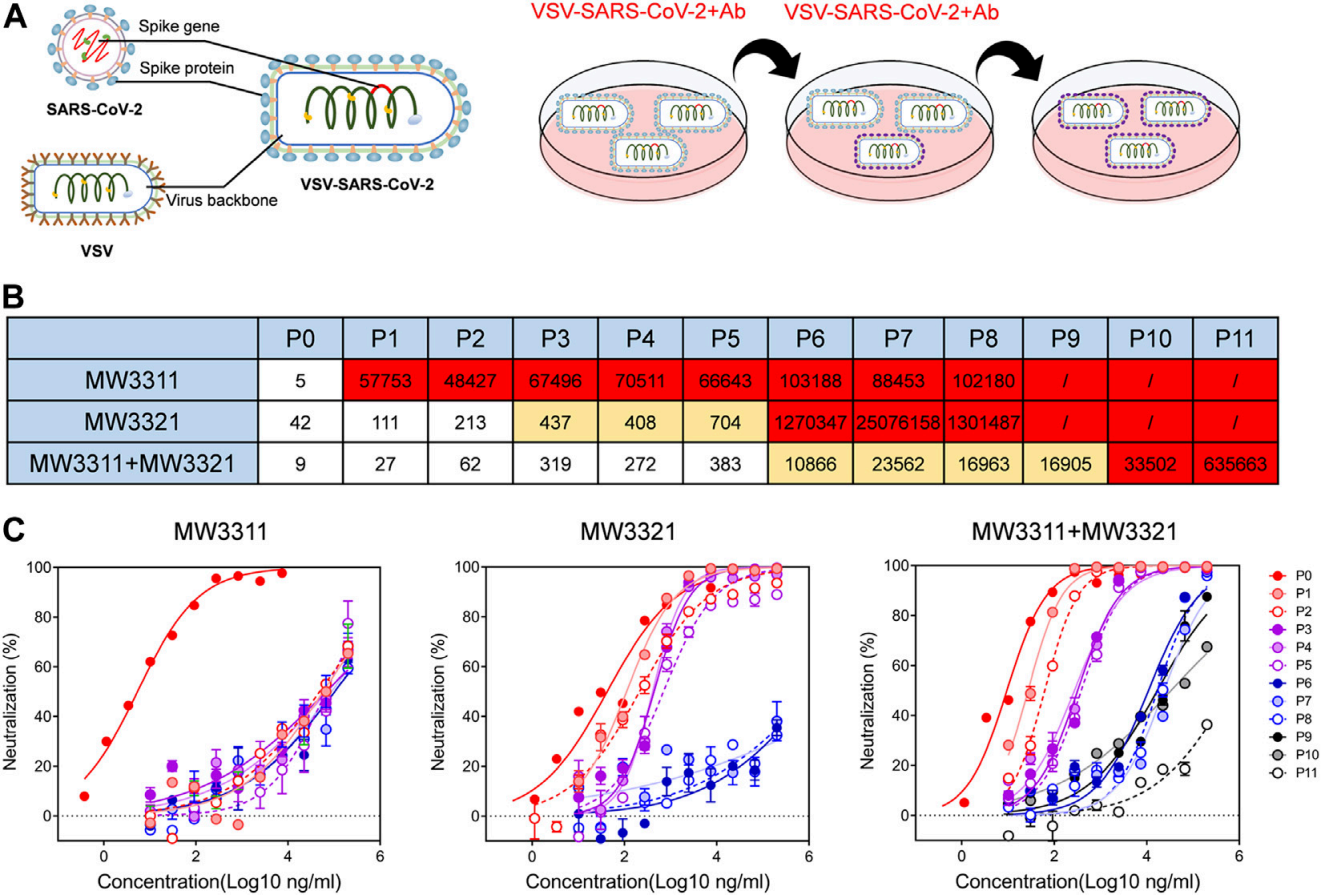
Escape mutation analysis of MW3321 is a SARS-CoV-2 pseudoviral system. **(A)** A schematic of the SARS-CoV-2 mutational escape study. Replicating VSV-SARS-CoV-2 is constructed based on the recombinant virus VSV-bearing spike protein (S) gene of SARS-CoV-2. VSV-SARS-CoV-2 was passaged under the additive antibody pressure. **(B)** Heatmap showing the antibody neutralization potency against VSV-SARS-CoV-2 virus cultured at each passage. The IC_50_ (μg/ml) value for each passage is shown with white, yellow, and red, indicating no escape (IC_50_ < 400 ng/ml), partial escape (400 ng/ml < IC_50_ < 2000 ng/ml), and complete escape (IC_50_ > 2000 ng/ml), respectively **(C)** Neutralization curves of MW3311 (left panel), MW3321 (middle panel) and the cocktail of MW3311 with MW3321 (right panel). The data points are represented as mean ± SEM with *n* = 2.

Apart from passively tracking emerged epidemic variants, escape mutation experiments could also be used to evaluate the resistance of MW3321 against potential mutations. The replicating SARS-CoV-2 pseudotyped virus, VSV-SARS-CoV-2-Sdel18, was used (Xiong et al., 2020) for escape mutation analysis, which consists of a G protein-deficient vesicular stomatitis virus (VSVdG) bearing a truncated spike protein (S with C-terminal 18 amino acid truncation), to mimic the evolutionary mutating of SARS-CoV-2 under the pressure of MW3321 (Figure 2A). MW3311, another SARS-CoV-2 neutralizing antibody, the epitope of which has no competition with that of MW3321(Jiang et al., 2021), and the combination of MW3321/MW3311 were also included in this experiment. Neutralization activity of pressure-antibodies against every passage of SARS-CoV-2 pseudovirus was determined and the IC_50_ was summarized in Figure 2B. The complete mutational escape is found on different passages under the pressure of different antibodies or antibody cocktails. MW3311 showed significantly reduced susceptibility to the mutating viruses appearing in passage 1 (Figures 2B, C). Notably, the generation of complete escape mutations under the pressure of MW3321 was delayed for several passages. The complete mutational escape was not generated until passage 6, which means MW3321 could prevent the rapid mutational escape of SARS-CoV-2 (Figures 2B, C). Moreover, the combination of MW3321 and MW3311 can even more effectively inhibit the escape of SARS-CoV-2 than every single component (Figures 2B, C). Together, MW3321 or the cocktail of MW3321 with another antibody with distinct epitopes could minimize the mutational escape of SARS-CoV-2.

### Therapeutic Efficacy of MW3321 Against Wild-Type and Delta SARS-CoV-2 in hACE2-Transgenic Mouse Model

**FIGURE 3:**
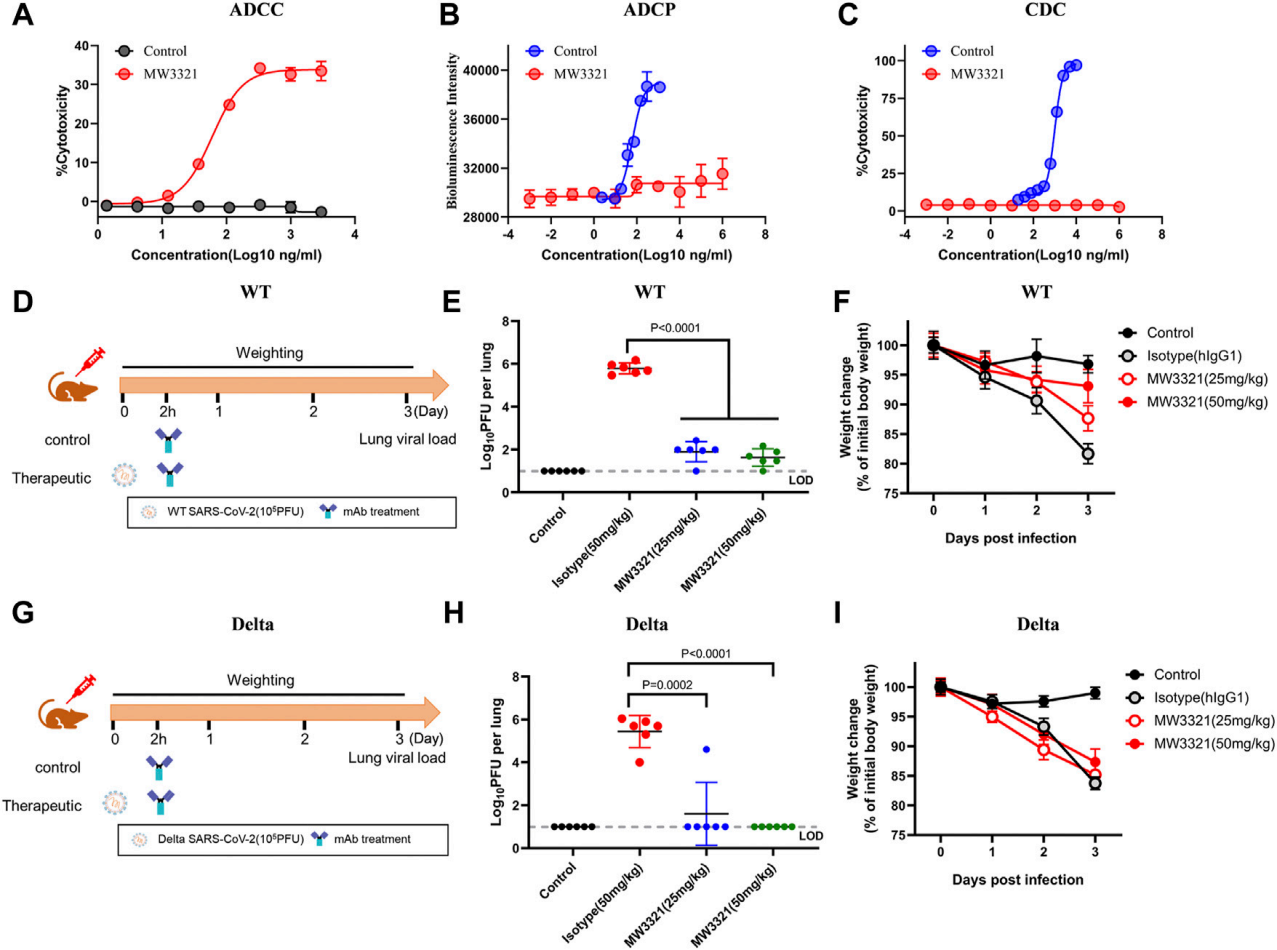
Therapeutic efficacy of MW3321 against SARS-CoV-2 wild-type and Delta strains in hACE2 transgenic mouse model **(A)** ADCC, **(B)** ADCP, and **(C)**CDC activity of MW3321 were assessed. The data points are represented as mean ± SD with *n* = 3. **(D,G)** Schematic diagram showing the *in vivo* experiment of MW3321. Mice were intranasally inoculated with 10^5^ pfu wild-type (D) or Delta (G) SARS-CoV-2. After viral challenge for 2 h, MW3321 (25 mg/kg or 50 mg/kg) or control antibody (50 mg/kg) were administered by intraperitoneal injection. The body weights of all animals were recorded daily and lung samples were collected and measured at 3 dpi. Uninfected mice and the infected mice treated with control antibodies (50 mg/ml) were used as control. **(E,H)** Viral burden in the lung samples of all animals was measured at 3 dpi. Dash lines indicate the limit of detection (LOD) of the assay. Error bar indicates SD of replicates. *p*-value was calculated with the Students’ t-test. **(F,I)** Body weight changes of all animals were measured. The data points are represented as mean ± SD with *n* = 6.

Fc-mediated immune functions of mAbs could contribute to *in vivo* protection by promoting viral clearance and anti-viral immune responses (Bournazos and Ravetch, 2017; Hamdan et al., 2020). To evaluate whether MW3321 is capable of mediating effector function, we assessed antibody-dependent cellular cytotoxicity (ADCC), antibody-dependent cellular phagocytosis (ADCP), and complement-dependent cytotoxicity (CDC) activity of MW3321. As shown in Figure 3A, MW3321 promoted robust ADCC activity following incubation of SARS-CoV-2 spike protein-expressing CHO-K1 target cells with human peripheral blood mononuclear cells (PBMCs), but no ADCP or CDC effect was observed (Figures 3B, C). These results indicate that Fc-mediated effector functions of MW3321 may participate in viral control *in vivo*.

To further explore the therapeutic efficacy of MW3321 against SARS-CoV-2 wild-type and Delta SARS-CoV-2 strains, hACE2-transgenic mice were administered a single dose of 25 mg/kg, 50 mg/kg of MW3321, or 50 mg/kg of isotype control 2 h after viral challenge (10^5^ pfu/mouse) (Figures 3D, G). The body weight was recorded daily and the viral load was measured at 3 dpi. Treatment with MW3321 (25 mg/kg or 50 mg/kg) significantly reduced the viral load in the lungs of wild-type or Delta SARS-CoV-2 infected mice (Figures 3E, H). Notably, no viral replication was observed in all mice of the Delta SARS-CoV-2 infected group treated by MW3321, revealing that the virus was fully neutralized in the lung. In the wild-type SARS-CoV-2 infected group, MW3321 prevented the decrease of body weight in a dose-dependent manner (Figure 3F). However, beyond our expectation, in the Delta SARS-CoV-2 infected mice, the same trend of body weight loss was observed for MW3321 and control antibody-treated groups (Figure 3I). The limitation and deficiency of the transgenic model, or the higher pathogenicity of Delta SARS-COV-2 (Saito et al., 2022), might be the reasons for the divergence between the wild-type and Delta groups.

Collectively, these data demonstrate that MW3321 could trigger Fc-mediated immune functions *in vitro* and that its *in vivo* anti-viral activity may rely on both neutralization and effector functions.

### Pharmacokinetic and Safety Profile of MW3321

**FIGURE 4:**
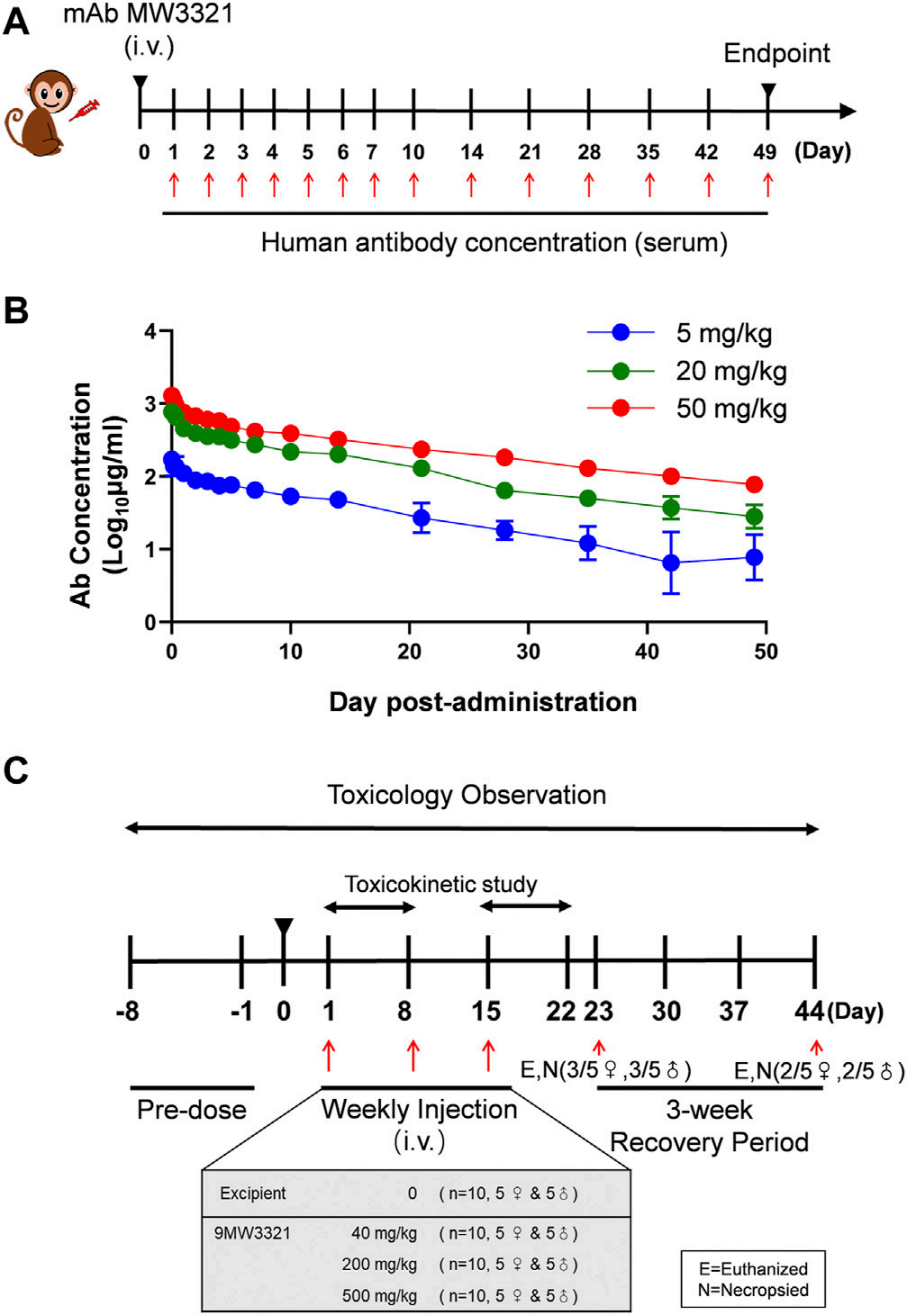
Pharmacokinetic profile of MW3321. **(A)** Schematic diagram of pharmacokinetic studies of MW3321. A single dose of MW3321 (5 mg/kg, 20 mg/kg, and 50 mg/kg) was administered to cynomolgus monkeys (3/sex/group) by intravenous injection. Serum samples were collected at different time points and measured after that **(B)** Mean serum MW3321 concentration in cynomolgus monkeys. Data are presented as mean ± SD, *n* = 6. **(C)** Schematic diagram of toxicology study. Different dose of MW3321 was administered to cynomolgus monkeys (5/sex/group) by intravenous infusions once a week for 3 weeks, with a 3-week recovery period. The indicated number of animals in each group were euthanized and necropsied on D23 and D44. Toxicokinetic studies started from pre-dose (0 h) to 168 h post-dose on D1 and D15. Other evaluations and parameters were studied during the pre-dose and post-dose periods.

The PK profile of MW3321 was characterized in a single-dose study in cynomolgus monkeys (Figure 4A). Following a single intravenous (i.v.) infusion of MW3321, no consistent gender differences in systemic exposure were noted. Both C_max_ and AUC_0-1176h_ increased basically with dose-proportionally over the dose range of 5–50 mg/kg. The range of average elimination half-life (T_1/2_) across dose levels was 292.4–417.3 h, clearance (CL) was 0.11–0.14 ml/h/kg, the volume of distribution (Vss) was 45.9–77.8 ml/kg, C_max_ was 178.8–1316.2 µg/ml, and AUC_0-240h_ was 41.1–313.0 h*mg/ml in this study. As the volume of monkey plasma is about 44.3–66.6 ml/kg, these results indicated that MW3321 was distributed mainly in the circulation system. Dose-independent terminal half-life and clearance were seen, indicating linear pharmacokinetic property within the dose range (Figure 4B). No anti-MW3321 antibody (ADA) was detected in any of the animals.

Repeated i.v. infusions of MW3321 to cynomolgus monkeys at 40, 200, or 500 mg/kg (QW×3) were well tolerated (Figure 4C). No unscheduled death or moribund sacrifice occurred during the course of the study. The only observed adverse reactions were small thymus and lymphocytopenia in the cortex in individual animals in all groups (Table 1). The decrease of cortical lymphocytes in the thymus in cynomolgus monkeys is related to aging as background lesions. The correlation between this finding and the test article may need further investigation. The NOAEL was considered as 40 mg/kg in this study. At this dose level, the mean C_max_ and AUC_0-168h_ on Day 15 were 2074 ± 429 µg/ml and 242715 ± 53173 h·mg/ml, respectively. The maximum tolerated dose (MTD) was determined to be 500 mg/kg. At this dose level, the mean C_max_ and AUC_0-168h_ on Day 15 were 24925 ± 5505 µg/ml and 2595818 ± 587202 h·mg/ml, respectively (Table 2).

**TABLE 1:** Toxicology study of MW3321.

	Evaluations	Effect of MW3321
Post-dose observations	Body weight	No effect
	Food consumption	No effect
	Body temperature	No effect
	Ophthalmoscopy	No effect
	Electrocardiogram	No effect
Clinical pathology	Hematology	No effect
	Serum chemistry	No effect
	Serum electrolyte	No effect
	Coagulation	No effect
	Urinalysis	No effect
Immunogenicity	ADA detection (ECL method)	No ADA detected
Immunotoxicity related tests	Lymphocyte Phenotyping: CD3^+^CD4+(%), CD3^+^CD8+(%), CD3^+^CD4+/CD3+CD8^+^	No effect
	Cytokines: IFN-γ, IL-2, IL-6, IL-8, IL-10, TNFα	No effect
	Circulating Immune Complex	No effect
	Others: WBC, TP, ALB, GLB, A/G, IgG, IgM, IgA, C3, and C4	No effect
Safety pharmacology	Cardiovascular system: blood pressure, systolic pressure, diastolic pressure, and electrocardiogram	No effect
	Respiratory system: respiratory frequency and tidal volume	No effect
	Functional observational battery (FOB): arousal, posture, gait, balance and co-ordination, slope, convulsions, tremors, myoclonus, general locomotor activity, grooming, lacrimation, piloerection, ptosis, retching/vomiting, saliva secretion, unusual behaviors/stereotypes, auditory startle response, urination and defecation, vocalizations, respiration, pupillary light response, blink reflex, ocular position/symmetry, pupil dimensions, and body temperature	No effect
Local tolerance	The administration site of each animal was marked, sampled, and fixed for histopathological analysis	No effect
Gross pathology and histopathology	Adrenal gland, aorta, sternum, brain, epididymis, esophagus, eye with optic nerve, fallopian tube, distal femur, gall bladder, heart, caecum, colon, duodenum, ileum, jejunum, rectum, kidney, liver, lung (with main stem bronchus), lymph nodes (axillary), lymph nodes (mesentery), mammary gland, sciatic nerve, ovary, pancreas, pituitary gland, prostate glands, salivary gland, mandibular, seminal vesicle, skeletal muscle, biceps femoris, skin, spinal cord (cervical, thoracic, and lumbar), spleen, stomach, testis, thymus, thyroid with parathyroid, trachea, urinary bladder, uterus with the cervix, vagina, gross lesions	small thymus and lymphocytopenia in cortex in individual animals in all groups
Organ weights and relative organ weights to terminal body weights and brain weights	Adrenal gland, brain, epididymis, heart, kidney, liver, ovary, spleen, testis, thymus, thyroid with parathyroid, uterus with cervix	No effect

**TABLE 2:** Main toxicokinetic parameters of MW3321.

Day	Parameter	Unit	Dose level		
			40 mg/kg(*n*=10)	200 mg/kg(*n*=10)	500 mg/kg(*n*=10)
D1	T_1/2_	h	228±102	221±106	157±72.5
	T_max_	h	0.684±1.25	1.09±1.66	2.67±3.39
	C_max_	μg/ml	1294±239	7003±1628	19428±4226
	AUC_0-168h_	h·μg/ml	113776±13361	570471±137334	1552253±198548
D15	T_1/2_	h	208±38.1	203±99.3	205±85.8
	T_max_	h	10.7±21.9	5.89±7.36	3.07±2.70
	C_max_	μg/ml	2074±429	10812±2729	24925±5505
	AUC_0-168h_	h·μg/ml	242715±53173	1155028±254725	2595818±587202

## Discussion

The increasing number of new SARS-CoV-2 variants with multiple mutations in the RBD represents a great concern regarding the effectiveness of current vaccines and antibody-based therapeutics. We previously reported that MW3321 shows strong cross-binding activity and high neutralizing potency with both SARS-CoV-2 and SARS-CoV. The binding epitope of MW3321 is highly conserved among SARS-related coronaviruses, which indicates the broad anti-viral activities of MW3321. In this study, we further proved that MW3321 remained high neutralizing potency against all tested 12 variants that have arisen in the human population. Some of these variants escaped neutralizing by some potent neutralizing antibodies, diminish the effectiveness of the proven vaccines, and reduce the efficiency of existing antibody cocktails (Wang et al., 2021a; Wang et al., 2021b; Hoffmann et al., 2021; Planas et al., 2021). Compared with wild-type SARS-CoV-2, MW3321 showed no obviously reduced susceptibility to other variants. Among these variants, no mutations appeared at the recognition site of MW3321, which further demonstrated the conservation of the epitope of MW3321. As of now, Omicron (B.1.1.529) has supplanted the Delta strain and become the dominant variant worldwide (Viana et al., 2022). MW3321 showed very weak neutralization activity to Omicron strain (data not shown). Omicron bears about 15 mutations in the RBD domain (McCallum et al., 2022), among which 4 mutations (S373P, S375F, N440K, and N501Y) are located in the epitope of MW3321. The considerable potential anti-viral activity of MW3321 against N501Y bearing Alpha, Beta, and Gamma reveals that N501Y doesn’t impact the potency of MW3321 (Figure 1). However, S375 is a main binding residue of MW3321 through several Hydrogen bonds and von der Waals interactions (Jiang et al., 2021), which may be a probable cause for the decreased neutralizing activity of MW3321 to Omicron.

The escape mutants screening study could offer predicted information about epitope and degree of evasion of SARS-COV-2 neutralizing antibodies (Baum et al., 2020; Li et al., 2022). Kyratsous’ group showed that under the pressure of a single antibody, like REGN10987 or REGN10933, the escape mutants were rapidly generated in P2 (Baum et al., 2020). In our study, MW3311 showed significantly reduced susceptibility to the mutating viruses appearing in passage 1 but retained a maximum neutralization up to ∼80% at high concentration, indicating weak prevention of mutational escaping. However, under the pressure of MW3321, complete escape was not generated until P6, revealing that MW3321 established a high barrier for the emergence of escape mutants. These pieces of evidence indicate that antibody targeting conserved epitope on Spike RBD would have the advantage of broad protection over other antibodies. The epitope of MW3321 will provide much information on the development of broadly protective therapeutics and vaccine designs against potential variants of SARS-CoV-2. In addition, a combination of MW3321 with other neutralizing antibodies like MW3311, further delayed the emergence of escape mutants (Figure 2), which might owe to the low possibility of formation of variants bearing multiple mutations to escape the neutralization of both antibodies, or deficiency of viral susceptibility, structural stability or replicating activity caused by the corresponding mutations, strongly supporting the important role of the combination of antibodies with distinct epitopes in the antiviral treatment.

MW3321 effectively protects against wild-type and Delta SARS-CoV-2 strains challenge *in vivo* in a hACE2 transgenic mouse model. In the meantime, MW3321 displays linear pharmacokinetic characteristics and that the half-life of MW3321 is in the range of what is expected for an IgG1 molecule in monkeys. In addition, the safety of MW3321 was further demonstrated in a GLP toxicology study using a cynomolgus monkey model where the MTD of MW3321 was 500 mg/kg. These combined evidence support further development of MW3321 for potential clinical use.

## Data Availability

The original contributions presented in the study are included in the article/supplementary material, and further inquiries can be directed to the corresponding authors.
